# RA-RAR signaling promotes mouse vaginal opening through increasing β-catenin expression and vaginal epithelial cell apoptosis

**DOI:** 10.1186/s12958-023-01084-8

**Published:** 2023-04-11

**Authors:** Nana Zheng, Wenbo Zhang, Xiaodan Zhang, Biao Li, Zhanying Wu, Yashuang Weng, Weiyong Wang, Jingjing Miao, Jing Yang, Meijia Zhang, Wei Xia

**Affiliations:** 1 Department of Reproductive Medicine Centre, Guangzhou First People’s Hospital, South China University of Technology, Guangzhou, Guangdong 510180 China; 2 State Key Laboratory for Agrobiotechnology, College of Biological Sciences, China Agricultural University, Beijing, 100193 China; 3 Division of Cell, Developmental and Integrative Biology, School of Medicine, South China University of Technology, Guangzhou, 510006 China; 4 School of Basic Medical Sciences, Shanxi Medical University, Taiyuan, Shanxi 030001 China

**Keywords:** Retinoic acid receptor, Vaginal opening, Apoptosis, β-catenin, Mouse

## Abstract

**Background:**

Retinoic acid (RA) plays important role in the maintenance and differentiation of the Müllerian ducts during the embryonic stage via RA receptors (RARs). However, the function and mechanism of RA-RAR signaling in the vaginal opening are unknown.

**Method:**

We used the *Rarα* knockout mouse model and the wild-type ovariectomized mouse models with subcutaneous injection of RA (2.5 mg/kg) or E2 (0.1 µg/kg) to study the role and mechanism of RA-RAR signaling on the vaginal opening. The effects of *Rarα* deletion on *Ctnnb1* mRNA levels and cell apoptosis in the vaginas were analyzed by real-time PCR and immunofluorescence, respectively. The effects of RA on the expression of β-catenin and apoptosis in the vaginas were analyzed by real-time PCR and western blotting. The effects of E2 on RA signaling molecules were analyzed by real-time PCR and western blotting.

**Results:**

RA signaling molecules were expressed in vaginal epithelial cells, and the mRNA and/or protein levels of RALDH2, RALDH3, RARα and RARγ reached a peak at the time of vaginal opening. The deletion of *Rarα* resulted in 25.0% of females infertility due to vaginal closure, in which the mRNA (*Ctnnb1, Bak* and *Bax*) and protein (Cleaved Caspase-3) levels were significantly decreased, and *Bcl2* mRNA levels were significantly increased in the vaginas. The percentage of vaginal epithelium with TUNEL- and Cleaved Caspase-3-positive signals were also significantly decreased in *Rarα*^*−/−*^ females with vaginal closure. Furthermore, RA supplementation of ovariectomized wild-type (WT) females significantly increased the expression of β-catenin, active β-catenin, BAK and BAX, and significantly decreased BCL2 expression in the vaginas. Thus, the deletion of *Rarα* prevents vaginal opening by reducing the vaginal β-catenin expression and epithelial cell apoptosis. The deletion of *Rarα* also resulted in significant decreases in serum estradiol (E2) and vagina *Raldh2/3* mRNA levels. E2 supplementation of ovariectomized WT females significantly increased the expression of RA signaling molecules in the vaginas, suggesting that the up-regulation of RA signaling molecules in the vaginas is dependent on E2 stimulation.

**Conclusion:**

Taken together, we propose that RA-RAR signaling in the vaginas promotes vaginal opening through increasing β-catenin expression and vaginal epithelial cell apoptosis.

**Supplementary Information:**

The online version contains supplementary material available at 10.1186/s12958-023-01084-8.

## Background

In mammals, the female reproductive tract provides a suitable site for mating, fertilization, embryo development, and fetus delivery [1], all of which are required for successful reproduction. A pair of Müllerian ducts fuses, elongates and differentiates into the oviduct, uterus, cervix, and upper vagina during prenatal development [2–4]. The uterovaginal canal extends to form the lower vagina during postnatal development [5, 6]. In mice, vaginal opening to the skin occurs at approximately 5 weeks of age through the tissue remodeling process [7], which is primarily composed of an estrogen-triggering apoptotic process in the epithelium of the distal vaginal cavity [8, 9].

Both the defects of Müllerian duct development and the failure of vaginal tissue remodeling result in a closed vaginal phenotype [1, 10]. Estrogen treatment advanced the time of complete vaginal opening [11], and estrogen signaling deficiency result in the closed vagina [1]. The closed vaginal phenotype has been observed in many genetically modified mice, in which the failure of the vaginal epithelial cell apoptosis prevents vaginal opening [8, 9, 12, 13]. Over-expression of anti-apoptotic gene Bcell lymphoma 2 (*Bcl2*) [7] or the double deletion of the pro-apoptotic genes *Bcl2*-associated X protein (*Bax*) and *Bcl2* antagonist/killer protein (*Bak*) [14] results in a closed vaginal phenotype in all adult females. The female mice with spontaneous point mutation in *Ctnnb1* (β-catenin^C429S^) exhibit unfused Müllerian ducts and no extended uterovaginal canal, resulting in double-lumen upper vagina and no lower vagina (closed vaginal phenotype) [10]. Moreover, overexpression of β-catenin could induce apoptosis [15], and β-catenin is involved in vaginal lumen formation [4]. The similar pathologies have been reported in patients without clear cause, such as vaginal atresia, vaginal septum and complex anomalies [16].

Retinoic acid (RA) is a biologically active metabolite of retinol (vitamin A) obtained from food [17]. RA is synthesized mainly by retinaldehyde dehydrogenase (RALDH) and degraded by cytochrome P450 enzyme (CYP26) [18]. RA regulates gene expression via binding to intracellular RA receptors (RAR), which heterodimerize with retinoid X receptors (RXR) to bind to RA response elements (RARE) [19]. RA-RAR signaling plays a variety of physiological processes including embryonic development, meiotic initiation, hematopoiesis, vision, cellular differentiation and apoptosis [20–24]. RA-RAR signaling also plays an imperative role in the maintenance and differentiation of the Müllerian ducts to form the female reproductive tract [25, 26]. The fetal rats with the lack of vitamin A exhibit incomplete development of the Müllerian duct, resulting in the absence of oviducts, uterus and vaginas [27]. Furthermore, the female mice of *Rarα*^*−/−*^*Rarβ2*^−/−^, *Rarα*^−/−^*Rxrα*^−/−^ or *Rarα*^−/−^*Rarγ*^−/−^ exhibit the absence of the Müllerian duct at the embryonic stage, and die during gestation or immediately after birth [22, 28]. In addition, previous studies indicated that vitamin A may act to prevent the irreversible stratification of the vaginal epithelium in neonatally estrogen-treated mice [29]. However, the effect of RA-RAR signaling on vaginal development at puberty has not been well investigated.

In this study, we focus on the function and molecular mechanisms of RA-RAR signaling during the vaginal opening. We find that RA signaling molecules are highly expressed in mouse vaginal epithelial cells at the time of vaginal opening. RA-RAR signaling increases β-catenin expression and vaginal epithelial cell apoptosis, resulting in the vaginal opening.

## Methods

### Animals and reagents

C57/BL6 mice were purchased from the Guangdong Medical Laboratory Animal Center (Guangzhou, China). *Rarα* heterozygous knockout mice on the C57BL/6J background were purchased from Gempharmatech Co.,Ltd (Nanjing, China). The mice were housed in individually ventilated cages at 22 ± 1 °C for a 12 h light-dark cycle and *ad libitum* access to water and food. *Rarα* homozygous knockout mice were obtained from the mating of *Rarα* heterozygous parental mice at 1:1. The day after partum was designated as 0.5 days postpartum (dpp). For genotyping, genomic DNA was isolated from tail biopsies by PCR with a combination of two primers. The primers were synthesized at BGI Genomics (BGI-Tech, Shenzhen, China) and the sequences are listed in Supplementary Table S1. The females were observed continuously daily afternoon from 17 dpp until the vaginal opening and recorded. To avoid male interference, monitored females were caged individually at 21 dpp. All experiments were conducted under the Guidelines of the Animal Care and Use Committee of South China University of Technology. Unless otherwise stated, the reagents were purchased from Sigma-Aldrich (St. Louis, MO, USA).

### RNA extraction and quantitative real-time PCR (RT-qPCR)

Total RNA was extracted from 2 mg of vaginal tissue or two adult ovaries using the ReliaPrep™ RNA Tissue Miniprep System (Promega, Madison, WI, USA). Then, the cDNA was reverse transcribed from 100 µg of total RNA using the GoScript™ Reverse Transcription System. RT-qPCR was carried out with a Light Cycler 96 instrument (Roche, Basel, Switzerland). The relative gene expression levels were calculated by the 2^−∆∆^Ct method. The relative quantity of mouse target genes normalized to ribosomal protein L19 (*Rpl19*). Each independent RT-qPCR experimental sample was derived from independent RNA extraction from different mouse vaginal and ovarian tissues. All the primer sequences used in this experiment are listed in Supplementary Table S2.

### Immunofluorescence and histologic analysis

For immunofluorescence, vaginal tissues were fixed in 4% paraformaldehyde (PFA), dehydrated, embedded in paraffin, and crosscut into 5 μm sections. Tissue sections were deparaffinized, rehydrated, and subjected to antigen retrieval with 0.01% sodium citrate buffer (pH 6.0) at high temperatures (95–98℃). Subsequently, the sections were cooled and blocked with 10% donkey serum for 1 h at room temperature, incubated with primary antibodies overnight at 4℃, and then incubated with Alexa Fluor 488- or 555-conjugated secondary antibodies (Thermo Fisher Scientific, Waltham, MA, USA) for 1 h at 37 °C. Finally, the sections were washed with phosphate buffered saline (PBS) and stained with 4′,6-diamidino2-phenylindole (DAPI) for 2 min. Sections were photographed using a Zeiss LSM 800 confocal microscope (Carl Zeiss, Oberkochen, Germany). The primary antibodies used are listed in Supplementary Table S3.

For histologic analysis, paraffin-embedded vaginal tissues from wild types (WT) and *Rarα* knockout females (*Rarα*^−/−^) were cut into 5 μm as described above. The sections were stained with periodic acid/Schiff reagent (PAS) and hematoxylin and examined by light microscopy.

### Western blotting

Total proteins from 2 mg of vaginal or spleen tissue, or two ovaries were extracted with WIP buffer (Cell Chip Biotechnology, Beijing, China) with 1 mM phenylmethylsulphonyl fluoride (PMSF) (Cell Signaling Technologies, Boston, MA, USA). The protein concentration was measured by the bicinchoninic acid (BCA) Protein Assay Kit (Beyotime, Shanghai, China), and 30 µg proteins per sample were separated by 10% sodium dodecyl sulfate (SDS)-polyacrylamide gel electrophoresis (PAGE). The protein bands were electrically transferred onto the PVDF membrane and blocked with 5% nonfat milk in Tris-buffered saline containing 0.1% tween (TBST) (pH = 7.6) on an orbital shaker for 1 h at room temperature, followed by incubation overnight at 4 °C with primary antibodies listed in Supplementary Table S3. Then, the membrane was washed with TBST for 30 min and incubated with matched secondary antibodies (1:5000) (Zhongshan Golden Bridge Biotechnology, Beijing, China) for 1 h at room temperature. The protein was visualized with SuperSignal West Pico Chemiluminescent Substrate (Thermo Fisher Scientific) and imaged by a Tanon 5200 chemiluminescent imaging system (Tanon, Shanghai, China). The band density was quantified by ImageJ software (NIH Image, Bethesda, MD, USA). Each independent western blotting experimental sample was derived from independent protein extraction from different mouse vaginal, spleen and ovarian tissues.

### Terminal deoxynucleotidyl transferase-dUTP nick end labeling (TUNEL) assays

In situ TUNEL assay was performed with Click-iT Plus TUNEL Assay (Thermo Fisher Scientific). The vagina sections from WT and *Rarα*^−/−^ females were fixed in 4% PFA for 15 min at 37℃ and digested with proteinase K for 15 min at room temperature. TUNEL assays were carried out conforming to the manufacturer’s instructions. In brief, the sections were incubated in a terminal deoxynucleotidyl transferase (TdT) reaction mixture for 60 min at 37℃, followed by treatment with Click-iT™ Plus TUNEL reaction cocktails containing Alexa Fluor 488 dyes for 30 min at 37℃ in the dark. Sections were counter stained with DAPI and the images were analyzed by a Zeiss LSM 800 confocal microscope (Carl Zeiss).

### Estradiol (E2) level assays

The WT and *Rarα*^−/−^ females at 30 dpp were anesthetized under diethyl ether, and whole blood samples were collected via cardiac puncture. Serum and blood cells were separated by static and centrifugation, and the serum E2 levels were measured by the estradiol radioimmunoassay kit (Beijing North Biotechnology Institute, Beijing, China).

### E2 and RA supplementation

E2 and RA (all-trans-retinoic acid, ATRA) supplementation was carried out with ovariectomy (OVX) mice. The 12-day-old females were anesthetized by intraperitoneal injection of 2.5% avertin, and then bilateral ovaries were excised. Ovariectomized female mice were then separated into the following groups: 17β-estradiol (E2, 0.1 µg/kg body weight. OVX-E2 group) vs. vehicle oil (OVX-oil group); RA (RA, 2.5 mg/kg body weight. OVX-RA group) vs. vehicle oil (OVX-oil group). The mice at 14 dpp were subcutaneously injected daily until 17 dpp. The female mice were sacrificed for RT-qPCR and western blotting analysis 24 h after the last injection.

### Statistical analysis

The experiments were performed at least three times. Data statistics were performed using SPSS 26.0 software (SPSS Inc., Chicago, USA) and graphs were performed using GraphPad Prism software (v8.3.0, La Jolla, CA, USA). The results are presented as the mean ± SEM. Two-tailed Student’s *t* test was used to analyze data between two groups. The one-way analysis of variance (ANOVA) followed by Dunnett post-hoc test was used to analyze data from more than two groups.

## Results

### The expression patterns of RA signaling molecules in the mouse vaginas

**Fig. 1:**
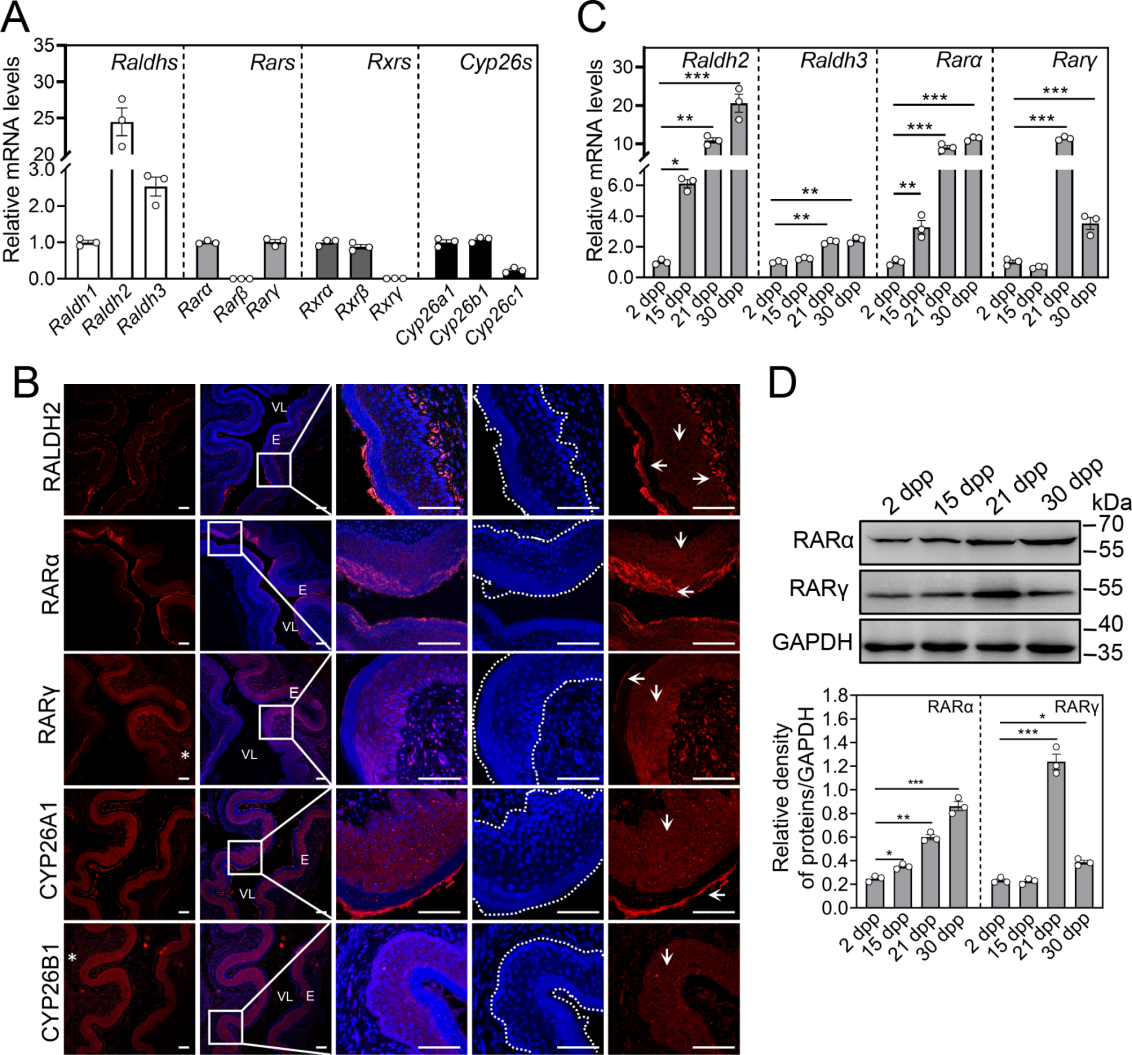
The expression pattern of RA signaling molecules in the vaginas. (**A**) The mRNA levels of *Raldhs*, *Rars*, *Rxrs* and *Cyp26s* in mouse vaginas at 5 weeks. The mRNA values of *Raldh1*, *Rarα*, *Rxrα* and *Cyp26a1* were set as 1, and those of other gens were normalized accordingly. (n = 3 independent experiments). Bars indicate the mean ± SEM. (**B**) Immunofluorescence staining for RALDH2, RARα, RARγ, CYP26A1 and CYP26B1 (red) in the vaginas at 5 weeks. The small white boxes indicate the enlarged areas as shown in the following images. Downwards arrows indicate epithelial cells of spinous layers, while leftwards and rightwards arrows indicate the epithelial cells of cornified layers and the stromal region near basement membrane layers, respectively. Asterisks (*) indicate endothelial cells. The nuclei were counterstained by DAPI (blue). The cells in the dashed white line box are vaginal epithelial cells. Scale bar, 100 μm. VL, vaginal lumen; E, epithelium. (**C**) The mRNA levels of *Raldh2*, *Raldh3, Rarα* and *Rarγ* in the vaginas at 2, 15, 21 and 30 dpp. The mRNA values of 2 dpp group were set as 1, and those of other groups were normalized accordingly. (n = 3 independent experiments). Bars indicate the mean ± SEM. *P < 0.05, **P < 0.01, ***P < 0.001 vs. 2 dpp group. (**D**) The protein levels of RARα and RARγ in the vaginas at 2, 15, 21 and 30 dpp. GAPDH was used as a loading control. (n = 3 independent experiments). Bars indicate the mean ± SEM. *P < 0.05, **P < 0.01 vs. 2 dpp group

First, we investigated the mRNA levels of RA synthesizing enzymes (*Raldh1, Raldh2*, and *Raldh3*), RA receptors (*Rarα, Rarβ*, and *Rarγ*), retinoid X receptors (*Rxrα, Rxrβ*, and *Rxrγ)* and RA catabolizing enzymes (*Cyp26a1, Cyp26b1*, and *Cyp26c1*) by RT-qPCR in the mouse vaginas at 5 weeks, in which vaginal opening occurred. The result showed that *Raldh2*, *Rarα*, *Rarγ*, *Rxrα*, *Rxrβ*, *Cyp26a1* and *Cyp26b1* were highly expressed (Fig. 1A). In the following studies, we chose these highly expressed isoforms for localization analysis. Immunofluorescence staining revealed that RALDH2 was strongly stained in the vaginal epithelial cells of the cornified and the stromal region near basement membrane layers, and weakly stained in the vaginal epithelial cells of spinous layers (Fig. 1B). RARα, RARγ and CYP26A1 were stained in the vaginal epithelial cells of the spinous and cornified layers, and RARγ was also stained in the endothelial cells (Fig. 1B). CYP26B1 was strongly stained in the vaginal epithelial cells of the spinous layers, and weakly stained in the endothelial cells (Fig. 1B).

Next, we compared the expression of RALDH2, RALDH3, RARα and RARγ in the mouse vaginas at 2–30 dpp. The mRNA and/or protein levels of RALDH2, RALDH3, RARα and RARγ were significantly increased from 2 to 30 dpp (Fig. 1C and D), and those of RARγ reached a peak at 21 dpp (Fig. 1C and D). These results suggest that the increases of RALDH2, RALDH3, RARα and RARγ expression levels in the mouse vaginas are positively related to the occurrence of the vaginal opening.

### *Rarα* deletion causes growth delay and progressive lethality

**Fig. 2:**
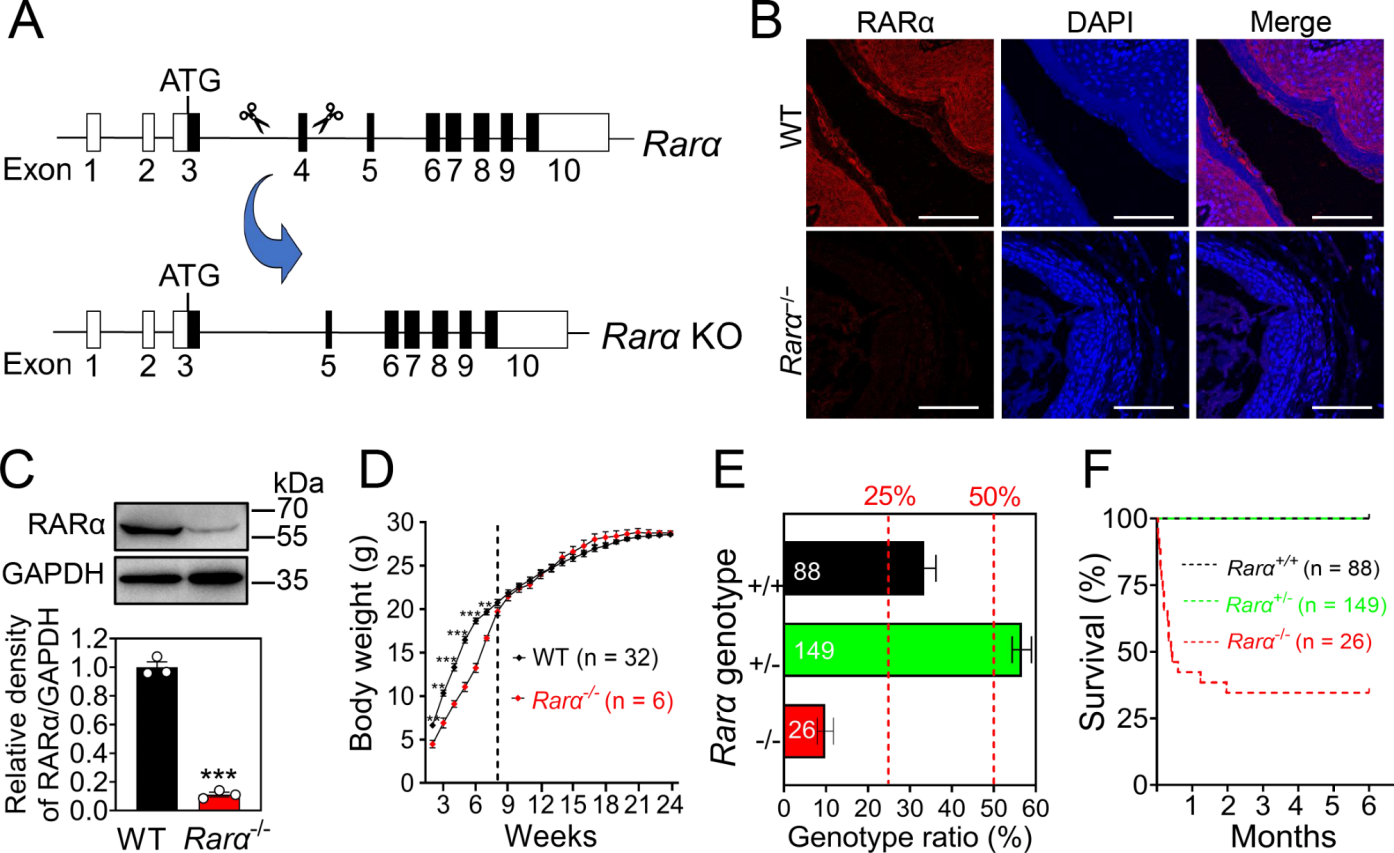
The effects of *Rarα* deletion on growth and survival. (**A**) The strategy of *Rarα* gene knock out (KO) by disrupting its exon 4 using CRISPR/Cas9 gene editing technology. (**B**, **C**) The detection of *Rarα* knockout efficiency in adult mouse vaginas by immunofluorescence (**B**) and western blotting (**C**) The nuclei were counterstained by DAPI (blue). Scale bar, 100 μm. GAPDH was used as a loading control. (n = 3 independent experiments.). Bars indicate the mean ± SEM. ***P < 0.001 vs. the WT group. (**D**) Body weight of *Rarα*^−/−^ female mice (n = 6) and their WT littermates (n = 32) from 2 to 24 weeks. Bars indicate the mean ± SEM. ***P < 0.001 vs. the WT group. (**E**) Frequencies of *Rarα* genotypes in offspring from 30 *Rarα*^+/−^ mating pairs were determined. Columns represent the percentage of the genotypes *Rarα*^+/+^ (black), *Rarα*^+/−^ (green), and *Rarα*^−/−^(red), and two dashed red lines indicate expected frequencies based on Mendelian inheritance. The pup number for each genotype is shown in the columns. (**F**) Survival curve of *Rarα*^+/+^ (black), *Rarα*^+/−^ (green), and *Rarα*^−/−^ (red) from the newborn to adulthood

To identify whether RA-RAR signaling was involved in regulating vaginal opening, we obtained the homozygous knockout mice (*Rarα*^*−/−*^) by crossing of heterozygote mice (*Rarα*^*+/−*^) with disrupted exon 4 of the *Rarα* gene on one chromosome (Fig. 2A). Genotyping was detected by common PCR (Fig. S1A). The *Rarα* knockout efficiency was confirmed by immunofluorescence and western blotting (Fig. 2B, C and Fig. S1B). Body size was comparable between *Rarα*^*−/−*^ and wild type (WT) mice (Fig. S3A). However, the body weight of *Rarα*^*−/−*^ females is significantly lower than WT mice from 2 to 7 weeks, but this difference disappeared after 8 weeks (Fig. 2D). The birth rate of *Rarα*^*−/−*^ mice was 9.9% (26/263), which was significantly lower than the expected Mendelian rate of 25.0% (Fig. 2E), suggesting *Rarα* deletion causes partly embryonic lethality. 57.7% of *Rarα*^*−/−*^ mice (15/26) died within 1 month of birth (Fig. 2F). These data demonstrate that *Rarα* deletion results in growth delay and progressive lethality.

### *Rarα* deletion could cause a closed vaginal phenotype

**Fig. 3:**
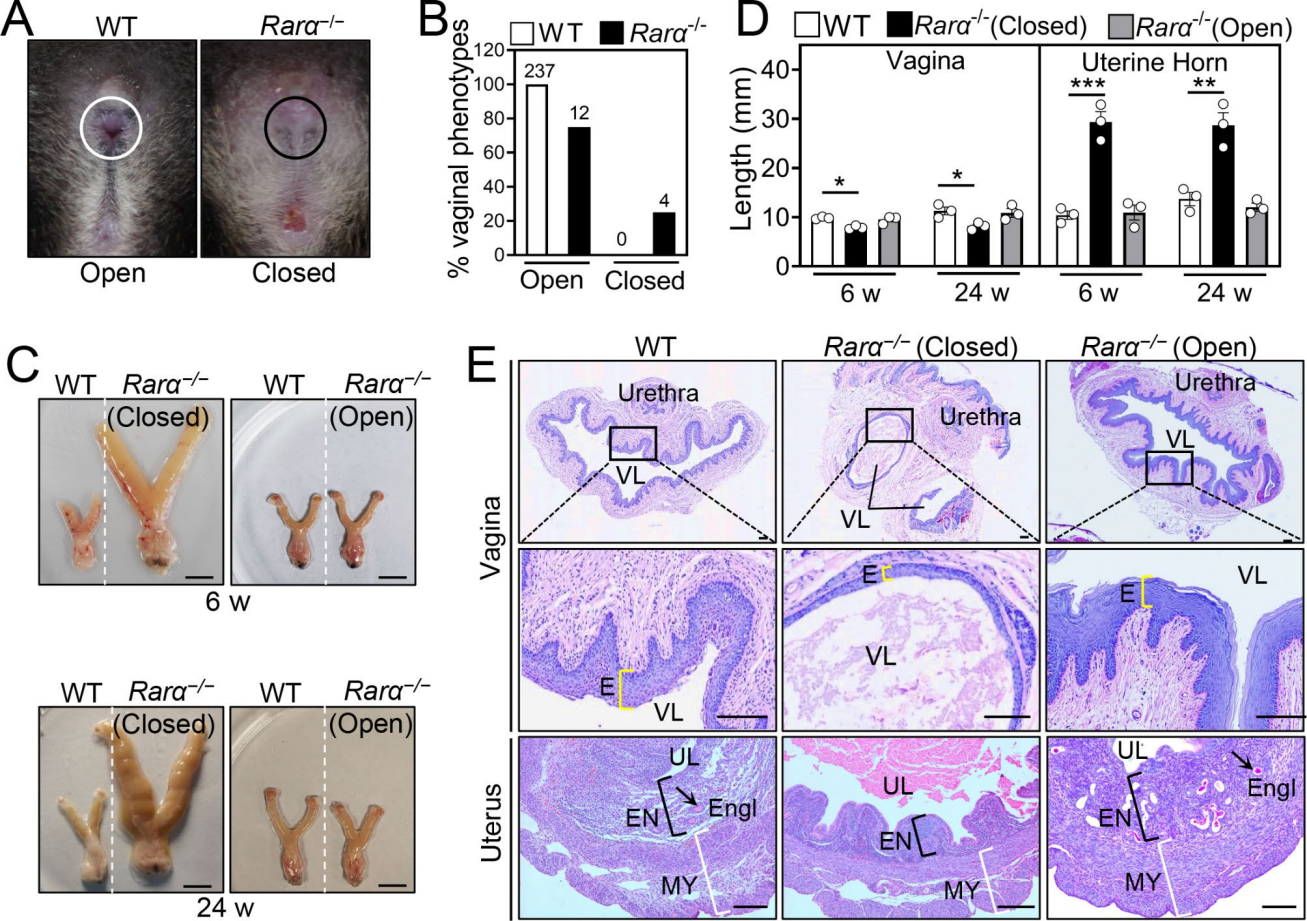
The effect of *Rarα* deletion on vaginal opening. (**A**, **B**) Representative pictures of the normal vaginal opening (white circle) in WT females and the vaginal closure (black circle) in *Rarα*^−/−^ females at 6 weeks (**A**), and the frequency of the closed vagina in *Rarα*^−/−^ females (**B**). The absolute numbers of females are indicated in the columns. (**C**, **D**) Representative female reproductive tracts (**C**) and the length of vagina and uterine horn (**D**) in *Rarα*^−/−^ females with vaginal open, *Rarα*^−/−^ females with vaginal closure and WT females at 6 and 24 weeks. Scale bar, 10 mm. (n = 3 independent experiments). Bars indicate the mean ± SEM. *P < 0.05, ***P < 0.001 vs. the WT group. (**E**) PAS-stained cross-sections for vaginas and uterus of *Rarα*^−/−^ females with vaginal open, *Rarα*^−/−^ females with vaginal closure and WT females. The small black boxes indicate the enlarged areas as shown in the following images. The yellow, black and white brackets indicate the thickness of vaginal epithelium (E), endometrium (EN) and myometrium (MY), respectively. Arrow, endometrial glands (Engl). Scale bar, 200 μm. VL, vaginal lumen; UL, uterine lumen

In WT and *Rarα*^+/−^ females, vaginal opening occurred around 30 dpp (Fig. S2). However, 25.0% of *Rarα*^−/−^ females appeared with a closed vaginal phenotype with the swelling of the genital area (Fig. 3A, B and Fig. S3B), which prevented natural mating and caused infertility. In the remaining *Rarα*^−/−^ female mice, the vaginal opening is normal (Fig. S2). Compared with WT females, the protein levels of RARγ had no change in the vagina of *Rarα*^−/−^ females with vaginal closure, but significantly increased in those of *Rarα*^−/−^ females with vaginal opening (Fig. S4). The *Rarα*^−/−^ females at 3 weeks exhibited small vagina (Fig. S3A) and the wet weight of the *Rarα*^−/−^ females (7.23 ± 1.67 mg; n = 3) was significantly lower than that of the WT mice (14.05 ± 0.91 mg; n = 4; *P* = 0.012). The *Rarα*^−/−^ females with vaginal closure exhibited enlarged vagina and uterus (Fig. 3C). Compared to WT, the vaginal length of the *Rarα*^−/−^ females with vaginal closure significantly reduced at 6 and 24 weeks (Fig. 3D), and the vagina usually had two lumens filled with fluid and the vaginal wall was thinner (Fig. 3E). In the *Rarα*^−/−^ females with vaginal closure, the uterine horn of was significantly lengthened (Fig. 3D), the endometrial and myometrial layers were obviously thin, and the endometrial gland was absent (Fig. 3E), which is likely to be secondary to the mechanical pressure exerted by fluid accumulation [30].

### *Rarα* deletion blocks vaginal epithelial cell apoptosis and decreases *Ctnnb1* expression

**Fig. 4:**
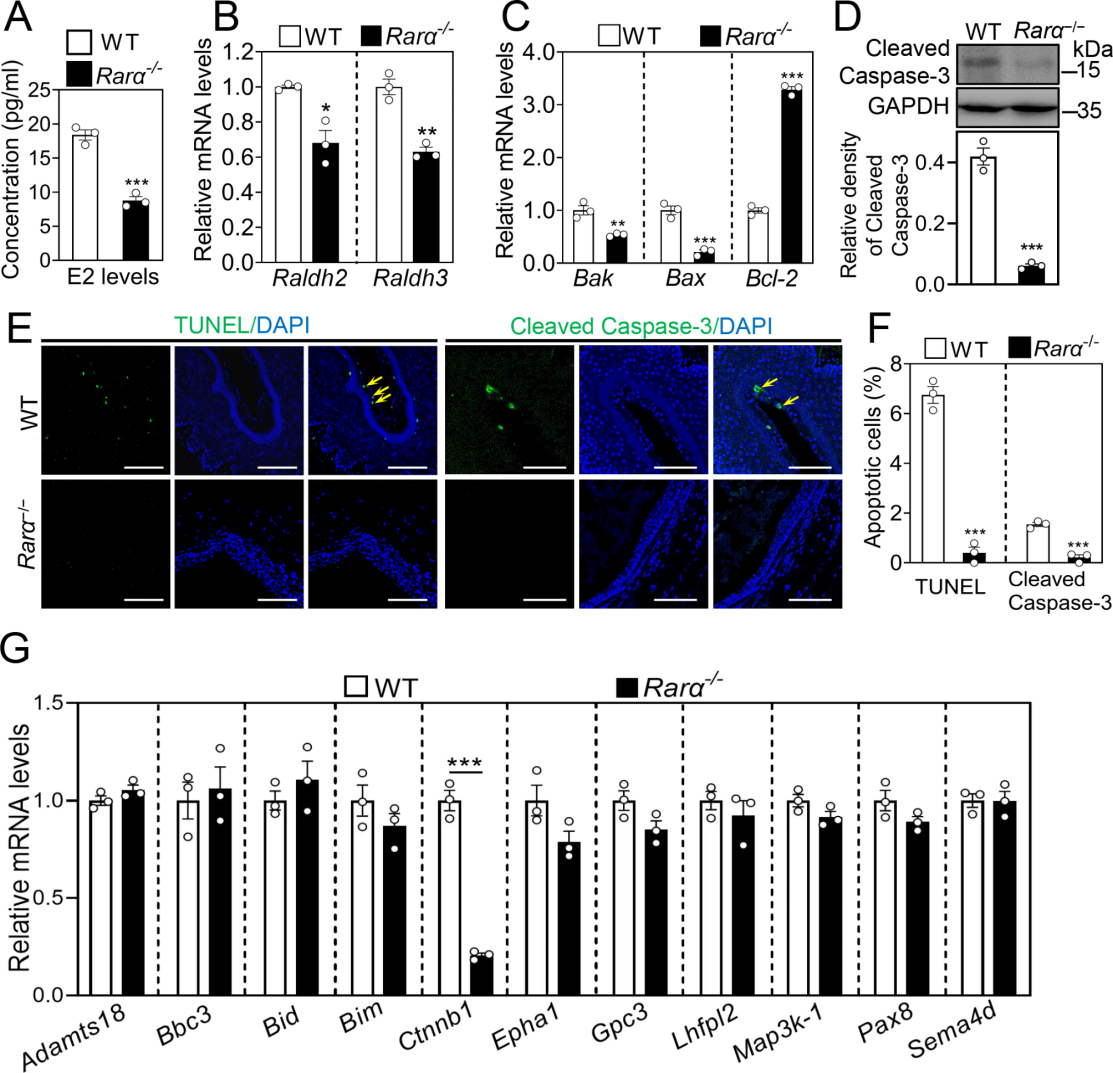
The effect of *Rarα* deletion on vaginal epithelial cell apoptosis and *Ctnnb1* mRNA levels. (**A**) The levels of serum estradiol (E2) in WT and *Rarα*^−/−^ females at 30 dpp. (n = 3 independent experiments). Data are presented as mean ± SEM. ***P < 0.001 vs. the WT group. (**B**, **C**, **D**) The mRNA levels of *Raldh2, Raldh3* (**B**), *Bak*, *Bax* and *Bcl-2* (**C**) and the protein levels of Cleaved Caspase-3 (**D**) in the vaginas of *Rarα*^−/−^ females with vaginal closure and WT females (non-estrus stage). The mRNA values of WT group were set as 1, and those of *Rarα*^−/−^ group were normalized accordingly. GAPDH was used as a loading control. (n = 3 independent experiments). Data are presented as mean ± SEM. *P < 0.05, **P < 0.01, ***P < 0.001 vs. the WT group. (**E**, **F**) Immunofluorescence stain of TUNEL and Cleaved Caspase-3 (green) (**E**), and the percentage of vaginal epithelial cells with TUNEL- and Cleaved Caspase-3-positive signals (green) (**F**) in each section in the vaginas of *Rarα*^−/−^ females with vaginal closure and WT females (non-estrus stage). The nuclei were counterstained by DAPI (blue). Yellow arrows, apoptotic cells. Scale bar, 100 μm. (n = 3 independent experiments. The representative images are shown). Data are presented as mean ± SEM. ***P < 0.001 vs. the WT group. (**G**) The mRNA levels of *Adamts18*, *Bac3, Bid, Bim, Ctnnb1*, *Epha1*, *Gpc3*, *Lhfpl2*, *Map3k1*, *Pax8* and *Sema4d* in the vaginas of *Rarα*^−/−^ females with vaginal closure and WT females. The mRNA values of WT group were set as 1, and those of *Rarα*^−/−^ group were normalized accordingly. (n = 3 independent experiments). Data are presented as mean ± SEM. ***P < 0.001 vs. the WT group

The vaginal opening process is largely dependent on massive vaginal mucosal apoptosis, which is initiated by rapidly elevated levels of estrogen at puberty [7]. The deletion of *Rarα* significantly decreased the serum estradiol (E2) levels (Fig. 4A) and the ovarian mRNA levels of *Cyp11a1*, *Cyp51*, *Ebp*, *Fdps*, *Msmo1*, *Mvk* and *Sqle*n (Fig. S5). Compared with the WT females, the vagina mRNA (*Raldh2, Raldh3, Ctnnb1, Bak* and *Bax* ) and protein (Cleaved Caspase-3) levels were significantly decreased (Fig. 4B and C), but the mRNA levels of the anti-apoptosis gene *Bcl-2* was significantly increased (Fig. 4C) in the *Rarα*^−/−^ females with vaginal closure. Consistent with these, the percentage of vaginal epithelial cells with TUNEL- and Cleaved Caspase-3-positive staining was significantly decreased in the *Rarα*^−/−^ females with vaginal closure. These results indicate that *Rarα* deletion decreases E2 levels and blocks vaginal epithelial cell apoptosis.

We next examined the mRNA levels of vaginal opening-related genes including *Adamts18*, *Bbc3*, *Bid*, *Bim*, *Ctnnb1*, *Epha1*, *Gpc3*, *Lhfpl2*, *Map3k1*, *Pax8* and *Sema4d*. Compared with WT females, only the mRNA levels of *Ctnnb1* were significantly decreased in the vaginas of *Rarα*^−/−^ females with vaginal closure (Fig. 4G). These results indicate that the decrease of β-catenin in the vaginas of *Rarα*^−/−^ females is involved in the closed vaginal phenotype.

### RA treatment promotes β-catenin expression and apoptosis progression in the vaginas

**Fig. 5:**
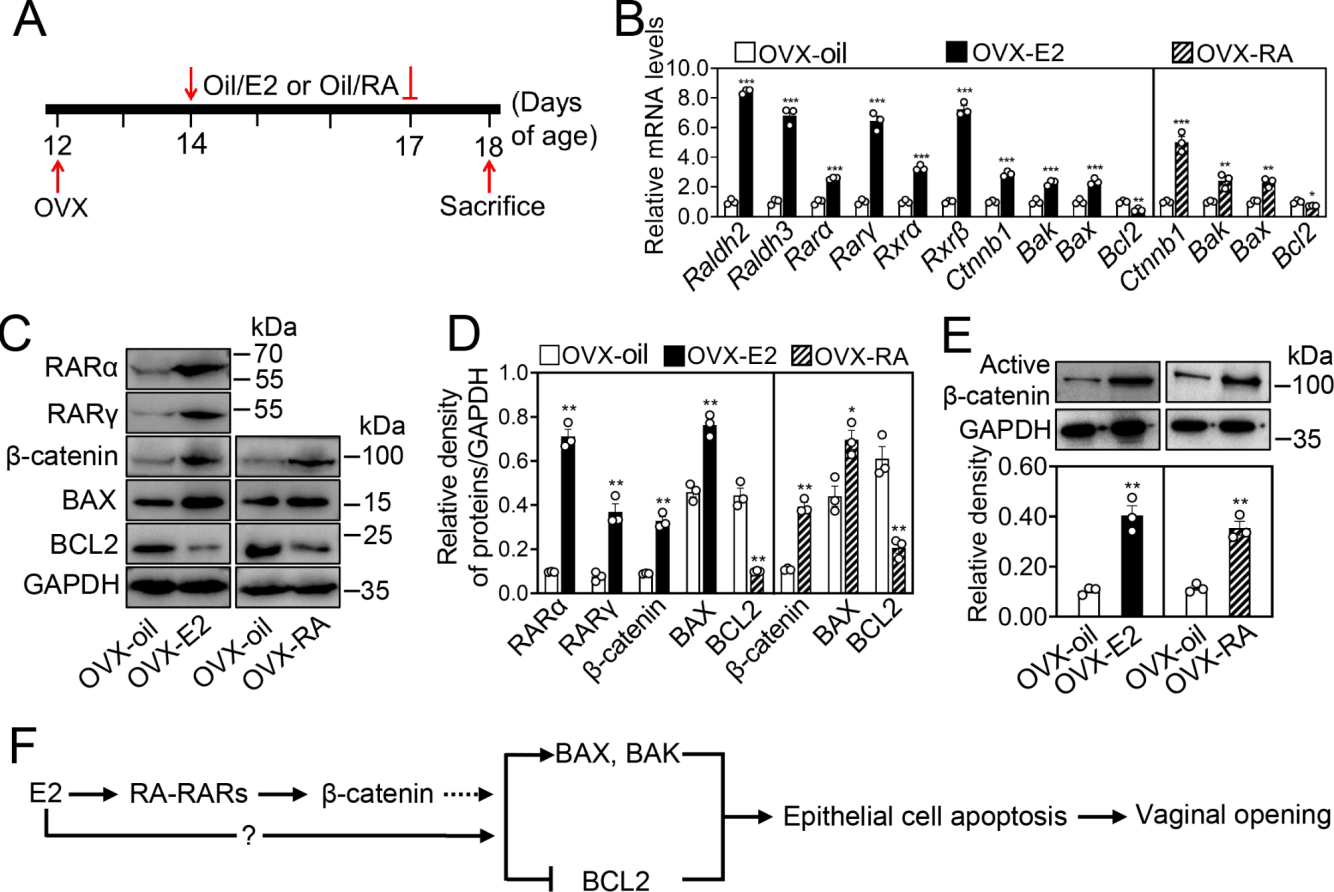
The effect of E2 and RA treatment on vaginal epithelial cell apoptosis and *Ctnnb1* mRNA levels in WT females. (**A**) The strategy of RA and E2 treatment in WT ovariectomized females. After ovariectomy (OVX), the females at 14 dpp received daily subcutaneous injections (s.c.) of E2 (0.1 µg/kg body weight. OVX-E2 group), RA (2.5 mg/kg body weight. OVX-RA group) or vehicle oil (OVX-oil group) until 17 dpp, and the females were sacrificed for experiments on vaginal tissue 24 h later. (**B**) The mRNA levels of *Raldh2*, *Raldh3, Rarα, Rarγ, Rxrα, Rxrβ, Ctnnb1*, *Bak*, *Bax*, and *Bcl2* in the vaginas of OVX-E2 and OVX-oil groups, and the mRNA levels of *Ctnnb1*, *Bak*, *Bax*, and *Bcl2* in the vaginas of OVX-RA and OVX-oil groups. The mRNA values of OVX-oil groups were set as 1, and those of other gens were normalized accordingly. (n = 3 independent experiments). Data are presented as mean ± SEM. *P < 0.05, **P < 0.01, ***P < 0.001 vs. the OVX-oil group. (**C**, **D, E**) The protein levels of RARα, RARγ, β-catenin, active β-catenin, BAK, BAX and BCL2 in the vaginas of OVX-E2 and OVX-oil groups, and the protein levels of β-catenin, active β-catenin, BAK, BAX and BCL2 in the vaginas of OVX-RA and OVX-oil groups. GAPDH was used as a loading control. (n = 3 independent experiments). Data are presented as mean ± SEM. *P < 0.05, **P < 0.01 vs. the OVX-oil group. (**F**) The proposed model for RA-RAR signaling in vaginal opening

RARs form heterodimers with RXRs and act as ligand-regulated transcription factors through binding specific RA response element sequences (RAREs, (A/G)G(G/T)TCA), which are usually located in the promoters of target genes [31]. RA promotes β-catenin expression in hippocampal neural stem cells [32]. Therefore, we analyzed the 1-2000 bp (bp) of the *Ctnnb1* promoter region and found three potential RARE-binding sites (Fig. S6A). The female mice at 12 dpp were initially subjected to ovariectomy, and then these females at 14 dpp were injected with RA (OVX-RA group) or with vehicle only (OVX-oil group. Figure 5 A). The mRNA levels of *Ctnnb1, Bak* and *Bax* were significantly increased, and those of *Bcl2* were significantly decreased in the vaginas of OVX-RA group contrast to OVX-oil group (Fig. 5B). Consistent with this, the protein levels of β-catenin and BAX were significantly increased, and BCL2 was significantly decreased in OVX-RA group (Fig. 5C and D). The protein levels of active β-catenin were also significantly increased (Fig. 5E). These findings indicate that RA signaling promotes β-catenin expression and activity and the apoptosis of the vaginal epithelium, ultimately triggering the vaginal opening.

It is reported that RA biosynthetic enzymes and receptors are up-regulated by E2 treatment in rat prostates [33]. We analyzed the 1-2000 bp (bp) in the promoter regions of *Raldh2*, *Raldh3* and *Rarα*, and found a potential estrogen response element sequence (EREs, AGGTCA) [34] in each of the promoter regions of these genes (Fig. S6B, C and D). Thus, we investigated the effect of E2 treatment on the expression of RA signaling molecules in the vaginas of ovariectomized females (OVX-E2 group. Figure 5 A). Compared with the OVX-oil group, the mRNA levels of *Raldh2, Raldh3, Rarα, Rarγ, Rxrα* and *Rxrβ* were significantly increased in the vaginas of OVX-E2 group (Fig. 5B). Consistent with this, the protein levels of RARα and RARγ were significantly increased in the vaginas of OVX-E2 group (Fig. 5C and D). Interestingly, the mRNA and protein levels of β-catenin, active β-catenin, BAK and/or BAX were also significantly increased, and those of BCL2 were significantly decreased in the vaginas of the OVX-E2 group in contrast to the OVX-oil group (Fig. 5B, C, D and E). These results suggest that E2 could promote vaginal opening by up-regulating RA signaling molecule expression in the vaginas.

## Discussion

Many genes are involved in vaginal opening in mice [1, 12, 35]. In the present study, we showed that the RA signaling molecules were significantly increased during the vaginal opening. RA supplementation of ovariectomized WT females significantly increased β-catenin, BAK and BAX expression levels in the vaginas, and the deletion of *Rarα* decreased β-catenin expression and vaginal epithelial cell apoptosis, resulting in the closed vaginal phenotype in 25.0% of females. Thus, RA-RAR signaling promotes mouse vaginal opening via inducing the vaginal epithelial cell apoptosis possibly by increasing β-catenin expression and activity.

Vaginal opening in mice at puberty is caused by vaginal epithelial cell apoptosis [8, 13]. Many genes (e.g., *Ctnnb1*, *Bak*, *Adamts18*) are involved in vaginal opening identified by genetically modified mouse models [10, 13]. RA supplementation of ovariectomized WT females increased β-catenin, active β-catenin, BAK and BAX expression in the vaginas. The deletion of *Rarα* caused vaginal closure in 25.0% of females, in which the mRNA (*Ctnnb1, Bak* and *Bax*) levels and the vaginal apoptotic cell number were significantly decreased. β-catenin is activated by Wnt signal and play many physiology functions [36]. The overexpression of β-catenin can induce activation of the p53-p21WAF1 pathway to induce cell apoptosis [15]. Thus, RA-RAR signaling may induce vaginal epithelial cell apoptosis by activating β-catenin pathway.

Mouse postnatal vaginal opening process is initiated by rapidly elevated estrogen to induce massive vaginal epithelial cell apoptosis [7, 37]. The deletion of *Rarα* also decreased serum E2 levels possibly by the decrease in steroidogenesis [38], which is involved in vaginal closure. Previous studies show that vitamin A inhibits the irreversible cell proliferation and cornification of vaginal epithelium in neonatal estrogen exposure mice, indicating that RA signaling may act in the vaginal epithelia to maintain homeostasis [29]. In our study, E2 supplementation of ovariectomized WT females at prepuberty increased the expression of RA signaling molecules, β-catenin, active β-catenin, BAK and BAX in the vaginas. Thus, E2 up-regulates RA signaling molecule expression, and then promotes β-catenin expression and activity in the vaginas, which may be involved in vaginal opening by inducing vaginal epithelial cell apoptosis (Fig. 5F). On the other hand, E2 may stimulate the expression of apoptotic genes and vaginal opening directly or indirectly by other pathways (Fig. 5F).

Vaginal opening indicates the maturation of the genital tract development and the onset of puberty in female mice [9]. The mutations in various genes appear different incidences of a closed vaginal phenotype [12, 13]. In this study, we found that RA signaling molecules were expressed in vaginal epithelial cells and were involved in the pubertal vaginal opening. The deletion of *Rarα* only resulted in a 25.0% incidence of the closed vaginal phenotype. The protein levels of RARγ had no change in the vagina of *Rarα*^−/−^ females with vaginal closure but were significantly increased in that of *Rarα*^−/−^ females with vaginal opening. The increase of RARγ protein levels may compensate for the function of RARα in the mutant mice. The functional redundancies between RAR isoforms have been reported in many studies. *Rarα*, *Rarβ*, or *Rarγ* null mutant mice are viable, but the double null mutants lacking either two RAR isoforms show abnormalities in embryonic development and death or serious deformity [20, 22]. The *Rar*α mutant mice used in our study were knockout of exon 4 of *Rarα* (containing 10 exons). A previous study reports that the mice with the knockout of exon 8 of *Rarα* have no closed vaginal phenotype [39]. This may be because a relatively long peptide chain is produced in the mice to play the function of RARα during vaginal development.

RA-RAR signaling is required for Müllerian duct maintenance and development during the embryonic stage [22, 28]. The spontaneous point mutations in *Ctnnb1* (β-catenin^C429S^) caused the double-lumen vagina due to the unfused Müllerian ducts at E15.5 and the no lower vagina (vaginal closure) due to the defect of extending to the vulva after birth [10]. In our study, the *Rarα*^−/−^ females with vaginal closure appeared a double-lumen and shorter vagina, probably because of the decrease of β-catenin in the vaginas of mouse. The shorter vagina may be also involved in the closed vaginal phenotype. It is reported that a patient has the similar pathology of longitudinal vaginal septum, resulting in dyspareunia and obstruction [40]. Whether RA signaling is involved in the above process needs further study.

## Conclusions

In conclusion, this study indicates that RA-RAR signaling, up-regulation by E2, promotes mouse vaginal opening through increasing β-catenin expression and vaginal epithelial cell apoptosis. Our findings have potential clinical implications for the diagnosis and treatment of vaginal developmental defects.

## Electronic supplementary material

Below is the link to the electronic supplementary material.

Supplementary Material 1

## Data Availability

The datasets used and/or analyzed during the current study are available from the corresponding author on reasonable request.

## Abbreviations

E2: estradiol
PFA: paraformaldehyde
DAPI: 4′,6-diamidino2-phenylindole
PAS: periodic acid/Schiff reagent
PMSF: phenylmethylsulphonyl fluoride
SDS: sodium dodecyl sulfate
PAGE: polyacrylamide gel electrophoresis
TBST: Tris-buffered saline containing 0.1% tween
WT: wild type
KO: knock out
RA: retinoic acid
RAR: retinoic acid receptor
RXR: retinoid X receptor
BAK: BCL2 antagonist/killer protein
BAX: BCL2-associated X protein
BCL2: B cell lymphoma 2
RALDH: retinaldehyde dehydrogenase
CYP26: cytochrome P450 enzyme
RARE: RA response element
TUNEL: terminal deoxynucleotidyl transferase-dUTP nick end labeling
ATRA: all-trans-retinoic acid
OVX: ovariectomy
ERE: estrogen response element
