# Supplementary material for: RA-RAR signaling promotes mouse vaginal opening through increasing β-catenin expression and vaginal epithelial cell apoptosis
Source: Reprod Biol Endocrinol. 2023 Apr 11;21:36. doi: 10.1186/s12958-023-01084-8 (PMC10088237; doi:10.1186/s12958-023-01084-8)
Supplement: Supplementary file 1 — Supplementary Material 1 [file 12958_2023_1084_MOESM1_ESM.docx]

**Supplemental Material**

Nana Zheng, et al.

**RA-RAR signaling promotes mouse vaginal opening through increasing β-catenin expression and vaginal epithelial cell apoptosis**

**Supplementary Figures**


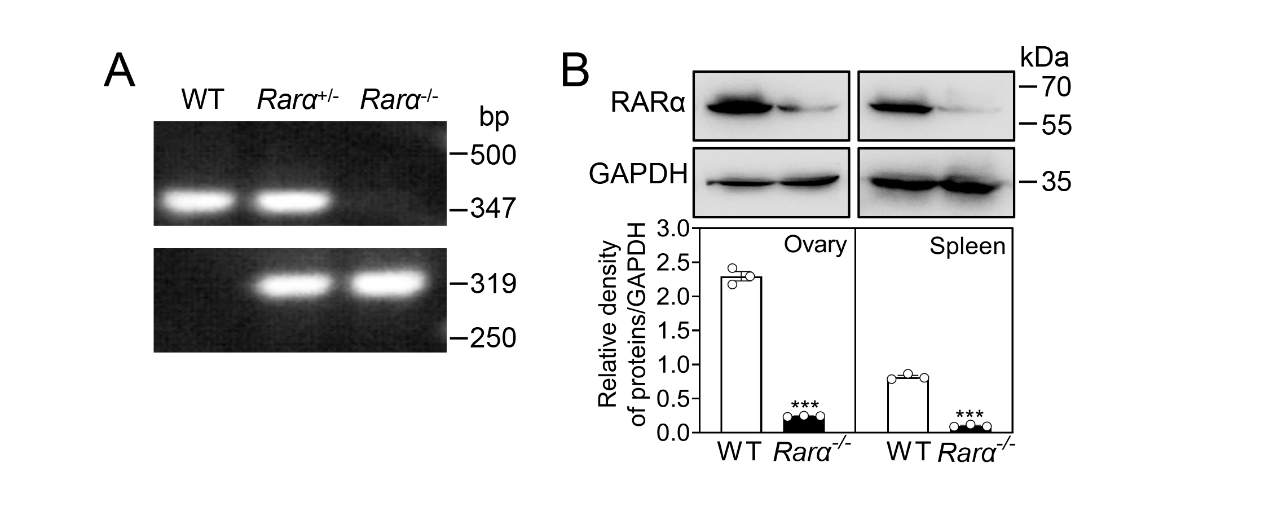


**Fig. S1** Detection of the genotype of *Rarα* mutants and knockout efficiency. (**A**) Common PCR analysis for genotypes of WT, *Rarα*^+/−^ and *Rarα*^−/−^. (**B**) Western blotting analysis for the knockout efficiency of RARα protein in ovaries and spleens. (n = 3 independent experiments). Bars indicate the mean ± SEM. ****P* < 0.001 vs. the WT group.


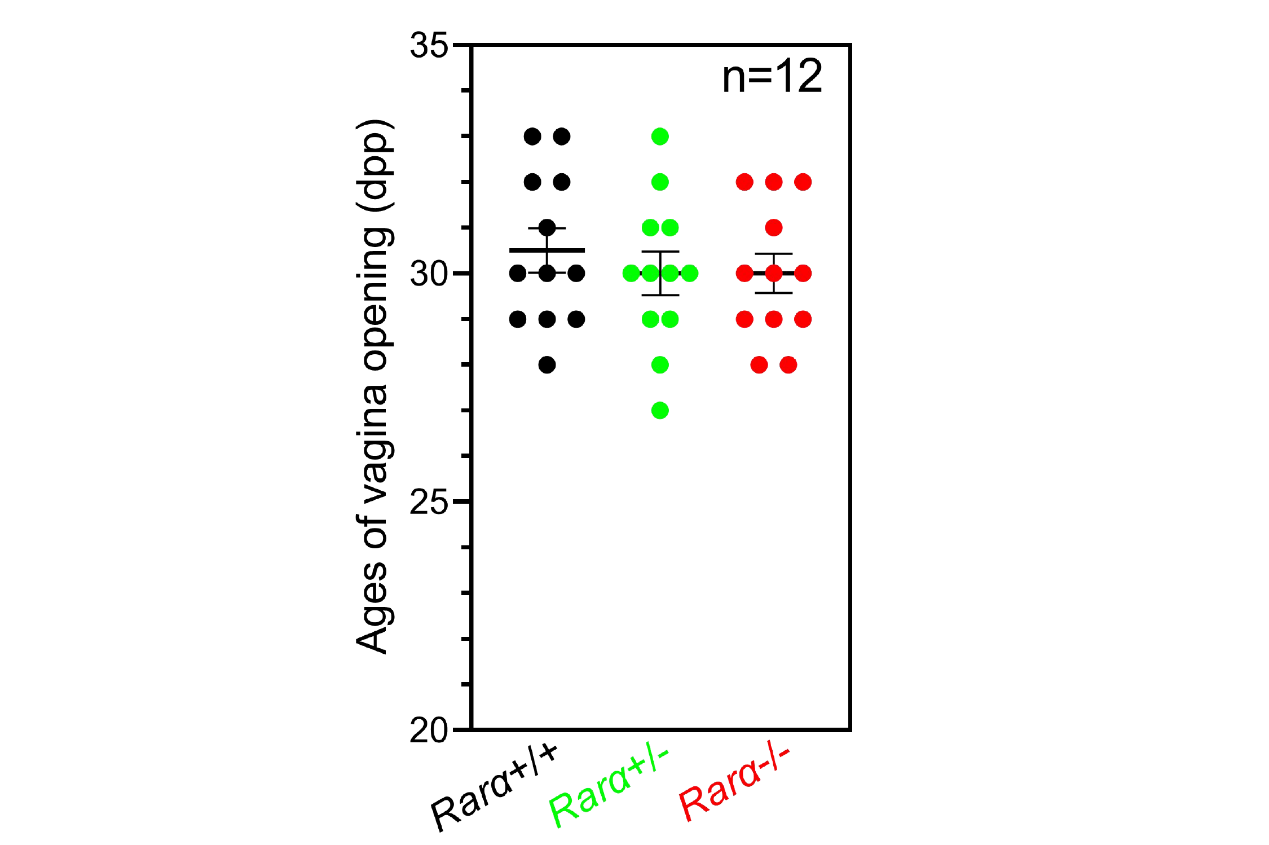


**Fig. S2** Detection of the vaginal opening ages of WT, *Rarα*^+/−^ and *Rarα*^−/−^ The average ages of vaginal opening of WT (black, 30 dpp), *Rarα*^+/-^ (green, 30 dpp), and *Rarα*^−/−^ (red, 30 dpp), showing no difference (n = 12). Bars indicate the mean ± SEM.


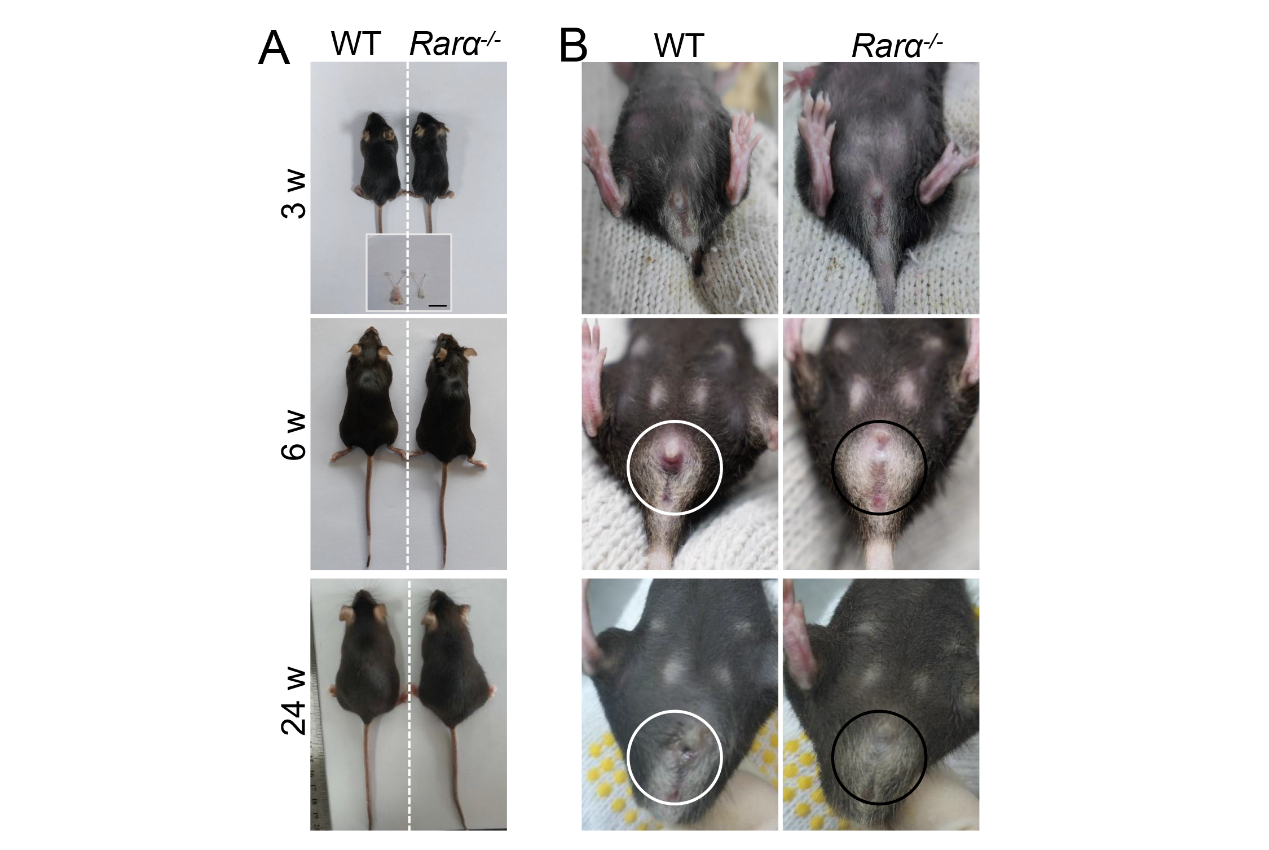


**Fig. S3** Uncropped images of vulvo-vaginal phenotypes of WT and *Rarα*^−/−^ mice. (**A**) Gross morphology of WT and *Rarα*^−/−^ mice at 3, 6 and 24 weeks and female reproductive tract of WT and *Rarα*^−/−^ mice at 3 weeks (white box). Scale bar, 10 mm. (**B**) The vulva-vaginal phenotype in WT mice and *Rarα*^−/−^ mice at 3 weeks, showing no difference; The normal vaginal opening (white circle) in WT mice, and vaginal closure and the swelling of the genital region in *Rarα*^−/−^ mice (black circle) at 6 and 24 weeks.


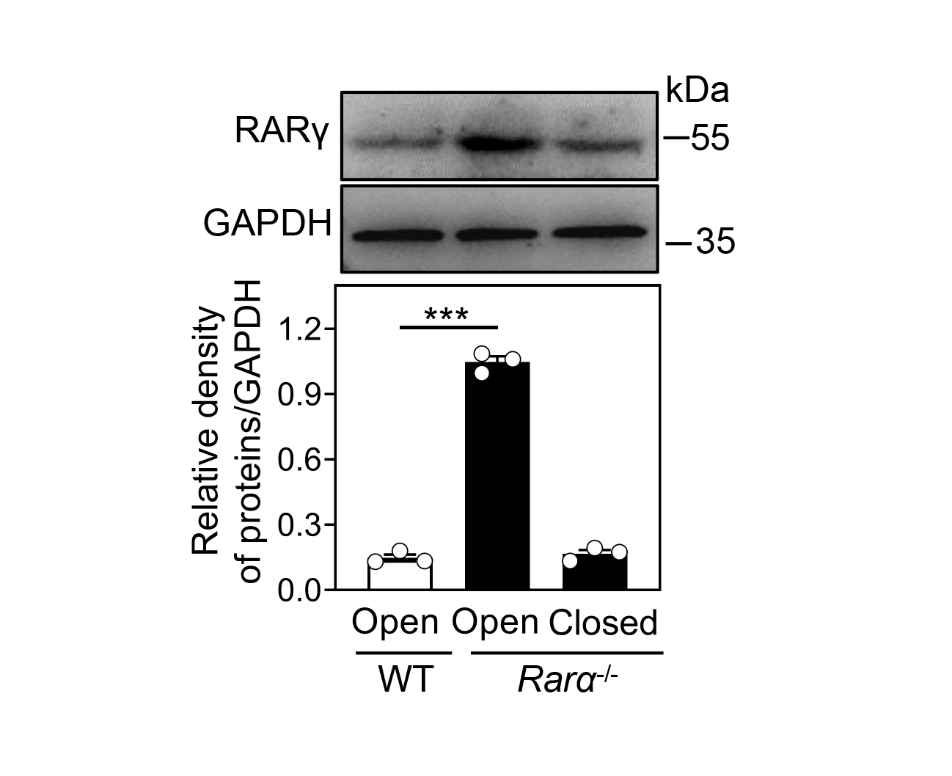


**Fig. S4** The effects of *Rarα* depletion on RARγ protein levels in the vaginas. The RARγ protein levels of *Rarα*^−/−^ females with vaginal closure, *Rarα*^−/−^ females vaginal with opening, and their WT littermates at 5 weeks. (n = 3 independent experiments). Bars indicate the mean ± SEM. ****P* < 0.001 vs the WT group.


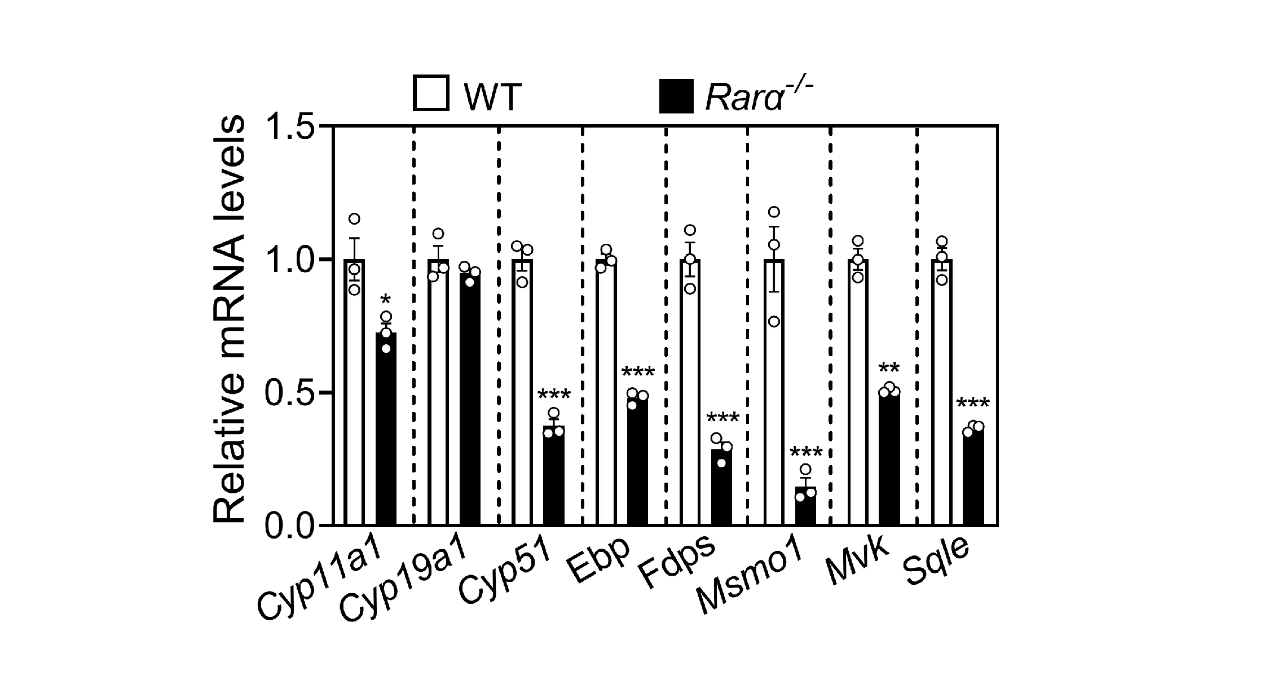


**Fig. S5** The effects of *Rarα* depletion on *Cyp11a1*, *Cyp19a1*, *Cyp51*, *Ebp*, *Fdps, Msmo1, Mvk* and *Sqle* mRNA levels in the ovaries. The mRNA levels of *Cyp51*, *Ebp*, *Fdps, Msmo1, Mvk* and *Sqle* in ovaries of *Rarα*^−/−^ females and their WT littermates at 5 weeks. Bars indicate the mean ± SEM. (n = 3 independent experiments). ****P* < 0.001 vs. the WT group.


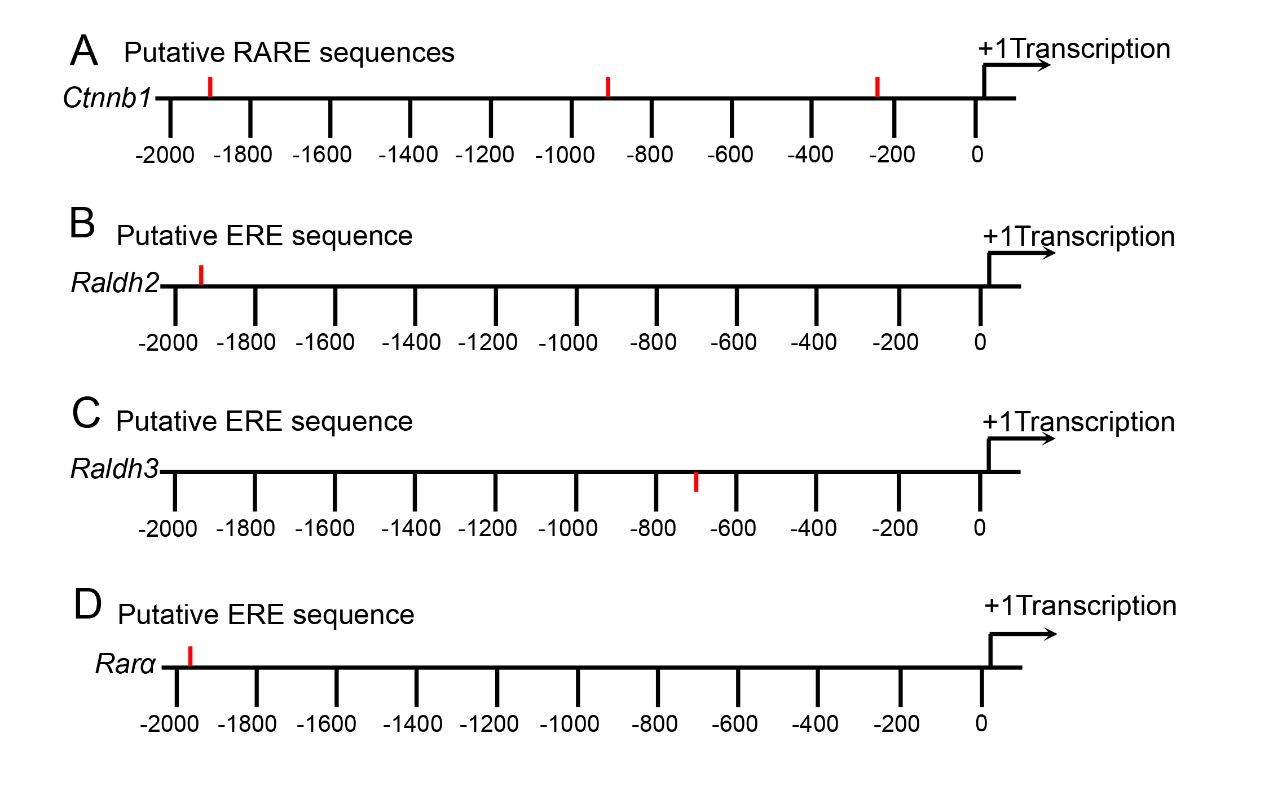


**Fig. S6** Schematic diagram of putative RARE sequences in *Ctnnb1* promoter and ERE sequence in *Raldh2/3* and *Rarα* promoters. (**A**-**D**) The mouse core promoters region of *Ctnnb1,* *Raldh2/3* and *Rarα*, 1-2000 bp before the 5′ untranslated region (UTR), were analyzed. (**A**) The red lines above the axis indicate putative RARE sequences, GGGTCA (−1901 bp to −1906 bp) and GGTTCA (−954 bp to −959 bp; −232 bp to −237 bp), in the *Ctnnb1* promoter. (**B**-**D**) The red line above indicates the putative ERE sequence, AGGTCA, in the *Raldh2* (−1926 bp to −1931 bp, **B**) and *Rarα* (−1962 bp to −1967 bp, **D**) promoter, and the red line below the axis indicates putative ERE sequence (−660 bp to −665 bp) in the *Raldh3* promoter complementary chain (**C**).

**
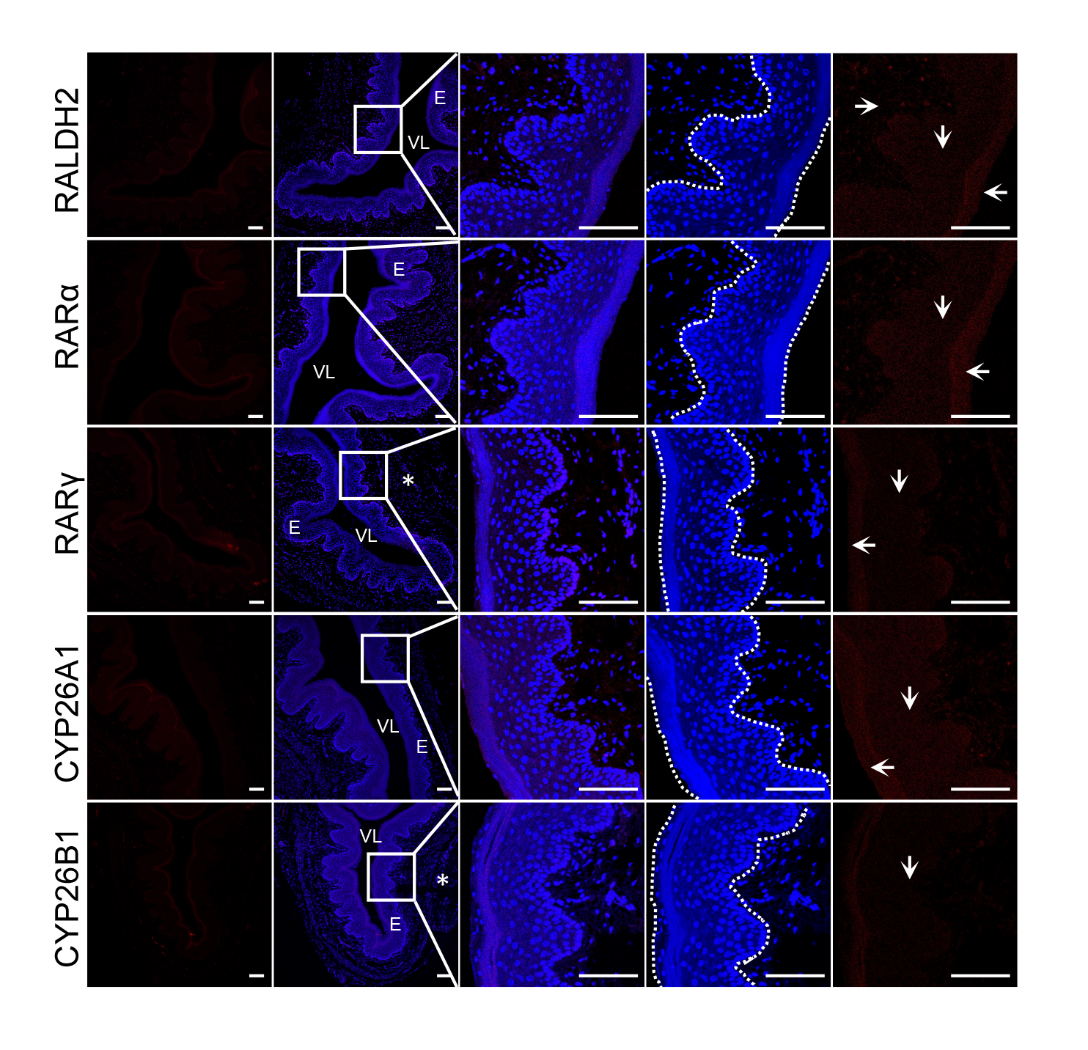
**

**Fig. S7 Negative control for Figure 1B.** Scale bar, 100 μm.

**
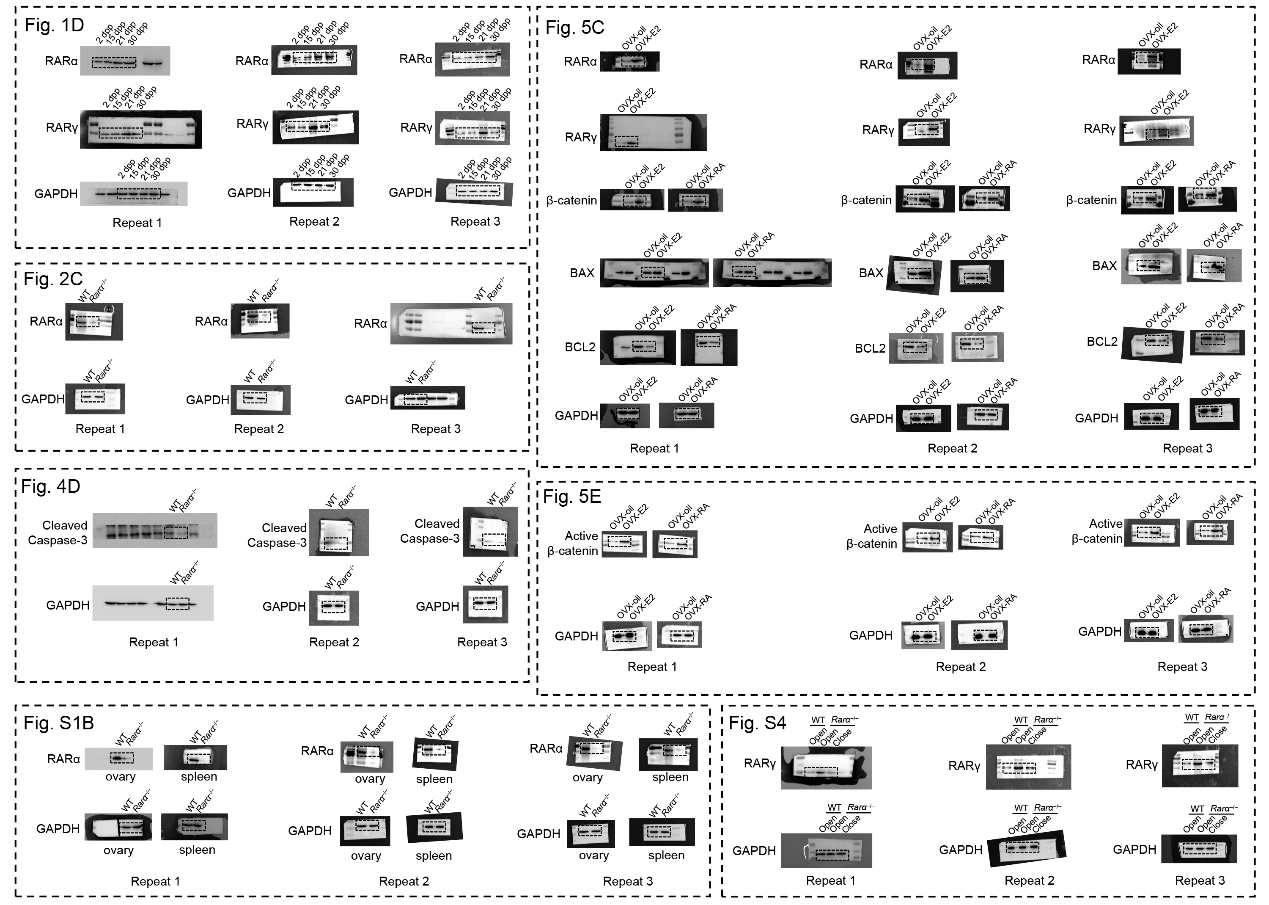
**

**Fig. S8 Uncropped scans of the western blotting results**.

**
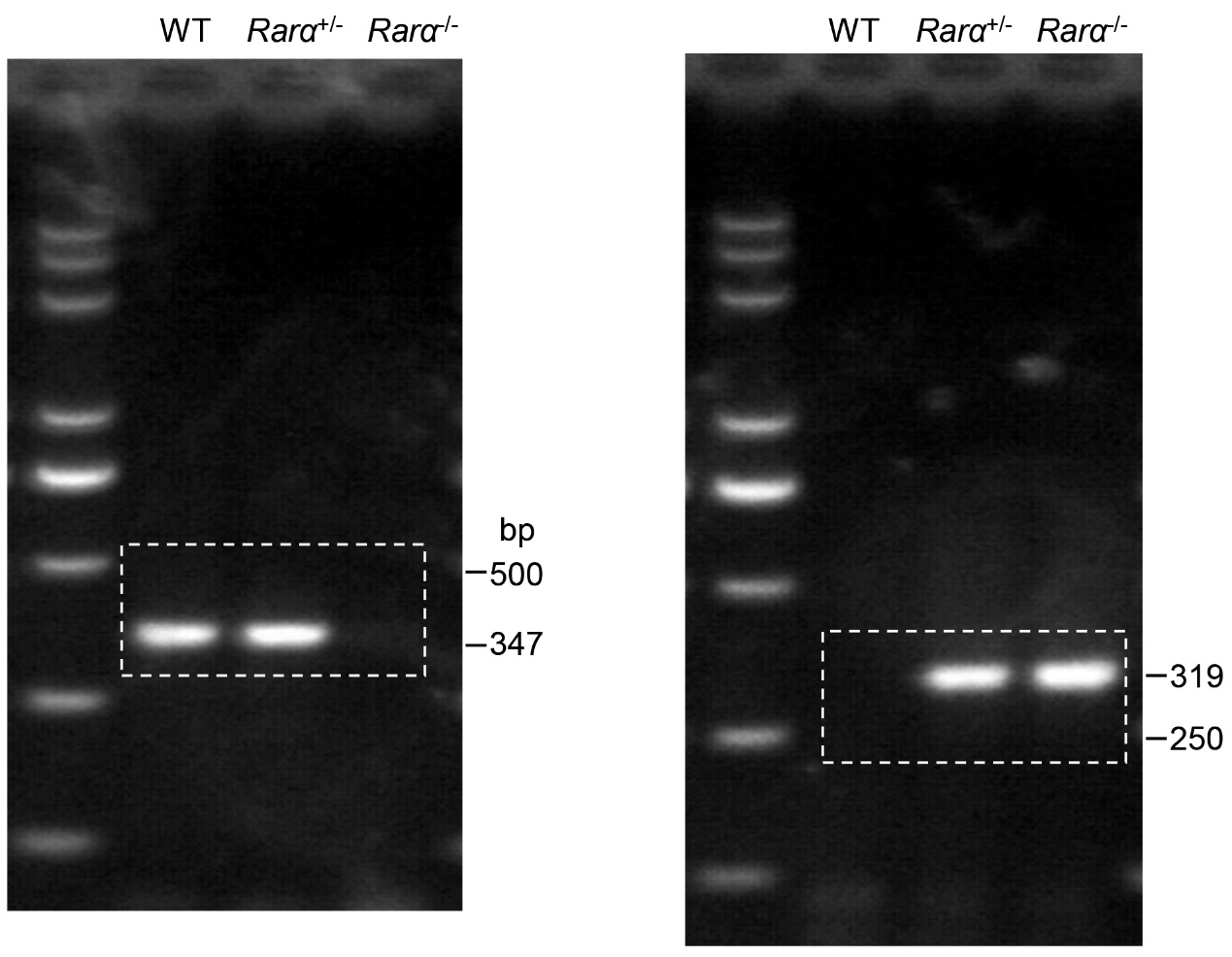
**

**Fig. S9 Uncropped scans of the agarose gel result**. PCR products from the mouse tail DNA of WT, *Rarα*^+/−^ and *Rarα*^−/−^ in the white dashed line box was used in Fig. S1A.

**Supplementary Tables**

**Table S1: Primers for genotype identification**

| **gene** | **Forward primer (5’-3’)** | **Reverse primer (5’-3’)** |
| --- | --- | --- |
| *Rarα-*KO | TAGTATGCAACCAGGCTCCTG | GAGACAAACACACTAGACAGGCAC |
| *Rarα-*WT | ATCTGGAATTGAAGCTGAGTCCTG | CATAGTGGTAGCCGGATGATTTG |

KO:319bp; WT: 347bp

**Table S2: Primers for RT-qPCR**

| **Genes** | **Forward primer (5’-3’)** | | **Reverse primer (5’-3’)** |
| --- | --- | --- | --- |
| *Adamts18* | GGTGGCAGCGGATTAAATGAT | | GGTGCAAAATGTCGTGTGAAATA |
| *Bak* | GCCCTGTACGTCTACCAGC | | TGGCGATGTAATGATGCAGTATG |
| *Bax* | TTTCATCCAGGATCGAGCAGG | | GCAAAGTAGAAGAGGGCAACCAC |
| *Bcl-2* | CTACCGTCGTGACTTCGCA | | TACCCAGCCTCCGTTATCC |
| *Ctnnb1* | AAGTGCAGTCTGTGTGATAAGG | | GACTCTCGGATGTGCAACCG |
| *Cyp26a1* | ACAAGACGCATCTGTTTGGG | | TGCATAATCACCTTCTTTCGCTG |
| *Cyp26b1* | TCATCGGAGAGACTGGTCACT | | GGTGCTCACTAGCTGGTGTTC |
| *Cyp26c1* | GCTACGGGACAGTGTTTAAGAC | | GCGCCAAGTAGTGTGTGTGA |
| *Cyp51* | ACTGGGGAATGGGAGATGGT | | AGGGCATGTCACAGAGTTGG |
| *Cyp19a1* | CATGGTCCCGCAAACTGTGA | | GTAGTAGTTGCAGGCACTTC |
| *Cyp11a1* | AGATCCCTTCCCCTGGCGACAATG | CGCATGAGAAGAGTATCGACGCATC | |
| *Ebp* | AGCACACAAAGGGCCAGAGT | | ACGGACTTGCCACCTTCTCA |
| *Fdps* | CGCGTTGAAGAACAGGGAGTG | | TGTAGGAAACCAAGCCACCTC |
| *Epha1* | GTGGACACTGTCATAGGAGAAGG | | GGTCTTAATGGCCACAGTCTTG |
| *Gpc3* | CAAGTCACTGCAAGTCACTCG | | CGGCCACAGTCCTTACTAAAC |
| *Lhfpl2* | ATGAGCAGGCAGGCATGAAC | | CAGAAGCCGCTGGCTATCTC |
| *Map3k1* | TAAATACCGGGTGTTTATTGGGC | | TTTTCTCCATAACATGGGGTCAG |
| *Msmo1* | CACAGACTCCTTCACCACAAGAGAA | | TTTCCAAGGGATGTGCGTATTC |
| *Mvk* | GCCTCCTTGGAACTCTCCCA | | CTTCTGAGACTTCTGCCCCG |
| *Pax8* | ATGCCTCACAACTCGATCAGA | | ACAATGCGTTGACGTACAACTT |
| *Raldh1* | TTAGCAGCAGGACTCTTCAC | | TTGGATAAGAACTGGGGTCA |
| *Raldh2* | GGAGGAGATCTTTGGTCCTG | | GCCAAACTCACCCATTTCTC |
| *Raldh3* | TCCATTTTATGCACAGGCTC | | CCATATCCTATCCGTCTGCC |
| *Rarα* | TTCTTTCCCCCTATGCTGGGT | | GGGAGGGCTGGGTACTATCTC |
| *Rarβ* | GCAGTGCGTGGACACATGA | | GGTAGAAGCTGCAAACTGACT |
| *Rarγ* | TGCCTGGTTTTACAGGGCTC | | TCCGAGAATGTCATAGTGTCCT |
| *Rxrα* | CACACCCACATTGGGCTTC | | GAGGCCATATTTCCTGAGGGA |
| *Rxrβ* | CCTACTCGTGTCGTGATAACAAA | | CTCAACGCCTTGGTCACTCT |
| *Rxrγ* | CATCTACACCTGTCGGGATAACA | | CATGTCTTCGTGGCTACTACTG |
| *Sqle* | ATCCTTCTACGCTCCCGACT | | GGACACGGGCCTCTCTATTG |
| *Rpl19* | CTGAAGGTCAAAGGGAATGTGTTC | | TGGTCAGCCAGGAGCTTCTTG |

**Table S3: List of antibodies**

| **Antibody** | **Catalog Code** | **Source** | **Host** | **Dilution** | |
| --- | --- | --- | --- | --- | --- |
|  |  |  |  | **IF** | **WB** |
| Active β-catenin | 05-665 | Millipore | Mouse |  | 1:500 |
| β-catenin | Ab32572 | Abcam | Rabbit |  | 1:1000 |
| BAX | 50599-2-Ig | Proteintech | Rabbit |  | 1:1000 |
| BCL2 | 26593-1-AP | Proteintech | Rabbit |  | 1:1000 |
| Cleaved Caspase-3 | 9664 | Cell Signaling Technology | Rabbit | 1:300 | 1:1000 |
| CYP26A1 | Ab151968 | Abcam | Rabbit | 1:500 |  |
| CYP26B1 | 21555-1 | Proteintech | Rabbit | 1:100 |  |
| RALDH2 | Ab75674 | Abcam | Rabbit | 1:200 |  |
| RARα | A19551 | ABclonal | Rabbit |  | 1:1000 |
| RARα | Ab28767 | Abcam | Goat | 1:200 |  |
| RARγ | LSB10760 | LifeSpan BioSciences | Rabbit | 1:200 | 1:1000 |
| GAPDH | 5174 | Cell Signaling Technology | Rabbit |  | 1:1000 |

IF: Immunofluorescence; WB: Western blotting
